# Supplementary material for: Pharmacogenetics and Adverse Events in the Use of Fluoropyrimidine in a Cohort of Cancer Patients on Standard of Care Treatment in Zimbabwe
Source: J Pers Med. 2023 Mar 28;13(4):588. doi: 10.3390/jpm13040588 (PMC10141018; doi:10.3390/jpm13040588)
Supplement: Supplementary file 1 [file jpm-13-00588-s001.zip › jpm-2244525-supplementary.pdf]

Pharmacogenetics and adverse events in the use of fluoropyrimidine in a cohort of cancer patients on standard of care treatment in Zimbabwe

Table S1: The frequency of Fluoropyrimidine-related adverse events during the total study period.

	Total study period, N=50		During the first 2 cycles, N=27	
	**Toxicities (≥3)**	**Toxicities (≤2)**	**Toxicities (≥3)**	**Toxicities (≤2)**
Treatment cycles, median (Range)	3 (2-6)	3 (2-6)	2	2
[a]Global	18 (36)	28 (56)	9 (33)	18 (66.6)
Gastrointestinal*	2 (4)	10 (20)	-	8 (29.6)
Nausea	2 (4)	7 (14)	-	7 (25.9)
Vomiting	-	9 (18)	-	7 (25.9)
Diarrhoea	1 (2)	4 (8)	-	1 (3.7)
Haematological*	16 (32)	25 (50)	9 (33)	14 (51.8)
Neutropenia	9 (18)	22 (44)	4 (14.8)	7 (29.6)
Leukopenia	4 (8)	13 (26)	2 (7.4)	5 (26)
Thrombocytopenia	2 (4)	8 (16)	-	3 (11.1)
Anaemia	7 (14)	17 (34)	5 (18.5)	9 (33.3)
HFS[b]	1 (2)	21 (42)	-	6 (22.2)

Dose reduction[c]	3 (6)	NA	1 (2)	NA
Discontinued treatment[d]	1 (2)	NA	-	NA

Abbreviations: HFS, hand-foot syndrome; NA, not applicable.

[a]Global includes all fluoropyrimidine-related AEs grade ≥3. This also includes dose reduction and treatment discontinuation.

*Gastrointestinal markers include nausea, vomiting, and diarrhoea.

*Haematological markers include neutropenia, leukopenia, thrombocytopenia, and anaemia

[b]HFS is defined as palmar-plantar erythrodysesthesia syndrome by the Common Terminology Criteria for Adverse Events version 5.0

[c]Dose reduction of fluoropyrimidines due to a fluoropyrimidine-related AE of grade ≥3.

[d]Patients discontinuing treatment with fluoropyrimidines due to a fluoropyrimidine-related AE of grade ≥3.

Table S2: Comparison of severe fluoropyrimidine adverse events with historical and literature cohorts.

	Sample size	Wild type Patients	DPYD Variant Carrier	Overall	Reference
[a]Global	50	18 (36)	0	18 (36)	(This study: Zimbabwe Cohort), 2023.
Gastrointestinal		2 (4)	0	2 (4)	
Haematological		16 (32)	0	16 (32)	
[a]Global	1394	418 (31)	11 (23)	429 (30.7)	(Wigle et al., 2021).
Gastrointestinal*		167 (12.4)	6 (12)	173 (12.4)	
Haematological*		157 (11.7)	6 (12)	163 (11.7)	
[a]Global	7365	1821 (33.6)	167 (50.2)	1888 (34.6)	(Meulendijks et al., 2015).

[a]Global includes all fluoropyrimidine-related AEs grade ≥3. It might not include dose reduction and treatment discontinuation.

*Gastrointestinal markers include nausea, vomiting, and diarrhoea.

*Haematological markers include neutropenia, leukopenia, thrombocytopenia, and anaemia

Table S3: Members of Consortium for Genomics and Therapeutics in Africa (CGTA)

SN	Name	Email	Country
1	Prof. Collen Masimirembwa	cmasimirembwa@aibst.edu.zw	Zimbabwe
2	Prof. Collet Dandara	collet.dandara@uct.ac.za	South Africa
3	Prof. Oluseye Bolaji	obolaji@oauife.edu.ng	Nigeria
4	Prof. Bernards Ogutu	ogutu6@gmail.com	Kenya
5	Dr. Ntokozo Ndlovu	ntokozosqo@gmail.com	Zimbabwe
6	Prof. Margaret Borok	mborok@gmail.com	Zimbabwe
7	Dr Patience Kuona	patiekuona@gmail.com	Zimbabwe
8	Prof. Jonathan Matenga	jonmatenga@gmail.com	Zimbabwe
